# Towards a Customizable, SLA 3D-Printed Biliary Stent: Optimizing a Commercially Available Resin and Predicting Stent Behavior with Accurate In Silico Testing

**DOI:** 10.3390/polym16141978

**Published:** 2024-07-11

**Authors:** Victoria Cordista, Sagar Patel, Rebecca Lawson, Gunhee Lee, Morgan Verheyen, Ainsley Westbrook, Nathan Shelton, Prakriti Sapkota, Isabella Zabala Valencia, Cynthia Gaddam, Joanna Thomas

**Affiliations:** 1 School of Engineering, Mercer University, Macon, GA 31207, USA; cordista.v@wustl.edu (V.C.); patel_sg@mercer.edu (S.P.); rebecca.lawson678@gmail.com (R.L.); gunhee.lee@live.mercer.edu (G.L.); verheyenmorgan@gmail.com (M.V.); ainsley.marie.westbrook@live.mercer.edu (A.W.); nshelt4@uic.edu (N.S.); prakriti.sapkota@live.mercer.edu (P.S.); isabella.zabalavalencia@live.mercer.edu (I.Z.V.); cynthia.s.gaddam@live.mercer.edu (C.G.); 2 McKelvey School of Engineering, Washington University, St. Louis, MO 63114, USA; 3 Medical College of Georgia, Augusta University, Augusta, GA 30912, USA

**Keywords:** biliary stent, 3D printing, SLA, sterilization, post-processing, FEA, CFD

## Abstract

Inflammation of the bile ducts and surrounding tissues can impede bile flow from the liver into the intestines. If this occurs, a plastic or self-expanding metal (SEM) stent is placed to restore bile drainage. United States (US) Food and Drug Administration (FDA)-approved plastic biliary stents are less expensive than SEMs but have limited patency and can occlude bile flow if placed spanning a duct juncture. Recently, we investigated the effects of variations to post-processing and autoclaving on a commercially available stereolithography (SLA) resin in an effort to produce a suitable material for use in a biliary stent, an FDA Class II medical device. We tested six variations from the manufacturer’s recommended post-processing and found that tripling the isopropanol (IPA) wash time to 60 min and reducing the time and temperature of the UV cure to 10 min at 40 °C, followed by a 30 min gravity autoclave cycle, yielded a polymer that was flexible and non-cytotoxic. In turn, we designed and fabricated customizable, SLA 3D-printed polymeric biliary stents that permit bile flow at a duct juncture and can be deployed via catheter. Next, we generated an in silico stent 3-point bend test to predict displacements and peak stresses in the stent designs. We confirmed our simulation accuracy with experimental data from 3-point bend tests on SLA 3D-printed stents. Unfortunately, our 3-point bend test simulation indicates that, when bent to the degree needed for placement via catheter (~30°), the peak stress the stents are predicted to experience would exceed the yield stress of the polymer. Thus, the risk of permanent deformation or damage during placement via catheter to a stent printed and post-processed as we have described would be significant. Moving forward, we will test alternative resins and post-processing parameters that have increased elasticity but would still be compatible with use in a Class II medical device.

## 1. Introduction

**Figure 1:**
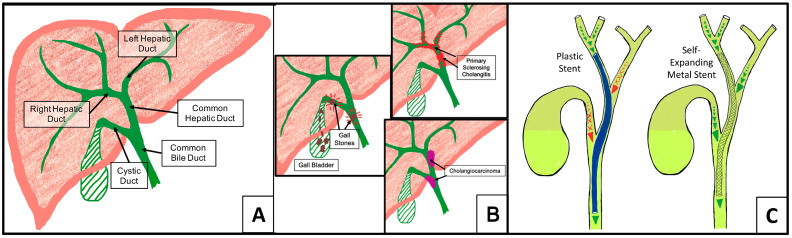
Hepatobiliary anatomy, cholestasis, and biliary stents. (**A**) The common hepatic duct merges with the cystic duct to form the common bile duct. Bile produced in the liver or stored in the gall bladder drains into the small intestine through the common bile duct. (**B**) Cholestasis caused by biliary strictures or obstructions is alleviated via palliative placement of plastic stents (**C**, left) or self-expanding metal stents (**C**, right).

Hepatocytes in the liver produce bile, which is funneled to the small intestine through bile ducts. Bile is stored in the gall bladder and released as needed to aid in the digestion of fats. When the extrahepatic bile ducts (EHBDs, Figure 1A) are inflamed or blocked, cholestasis, a condition where bile is unable to drain out of the liver, can occur (Figure 1B) [1,2]. Cholestasis is often caused by gallstones, primary sclerosing cholangitis (PSC), or cholangiocarcinoma [2,3,4] and is palliatively treated with self-expanding metal (SEM) or plastic biliary stents [5,6,7,8,9]. Both stent types have drawbacks. Plastic stents, while less expensive, can impede bile flow at bile duct junctures and frequently become clogged [10,11]; uncovered SEMs allow bile flow but can become embedded in the duct wall due to fibrotic tissue growth, and covered SEMs can migrate after placement [12] (Figure 1C).

Modified biliary stent architectures and/or materials could address these issues. As of 2023, only nine biliary stents were FDA-approved for use in the US: six SEM and three plastic stents [10,13,14]. The SEMs are chiefly made of nitinol; some have coatings to deter tissue ingrowth, while others are fully covered. Plastic stents are made of either polypropylene or polyethylene. Both SEMs and plastic stents can be deployed via percutaneous transhepatic cholangiogram or endoscopic retrograde cholangiopancreatography (ERCP). The latter requires a guide catheter that can vary in size from 6 F (2 mm) to 12 F (4 mm). Stents range in length from 2 cm to 25 cm. Plastic stents are 7 F to 11.5 F in diameter; SEMs expand up to 10 mm once deployed. While pre-clinical studies with Paclitaxel, Gemcitabine, Cisplatin, Dexamethasone, and/or Sorafenib have demonstrated improved outcomes [15,16,17,18,19,20], clinical trials with Paclitaxel-eluting biliary stents have shown no significant improvement over standard biliary stents [21,22]. More recent studies have focused on biodegradable polymeric biliary stents [16,23,24,25,26]. Typically made of a combination of polylactic acid (PLA), poly lactic-co-glycolic acid (PLGA), polycaprolactone (PCL), and/or polyethylene glycol (PEG), the degradation rates of these biliary stents can be adjusted to meet patients’ needs; observational clinical trials with biodegradable biliary stents are underway in the EU, Ecuador, and Asia as of May 2024 [27,28,29].

The improved resolution of 3D printers has made it feasible to 3D-print small, intricate devices such as biliary stents. As Class II medical devices, the material(s) and method(s) used to fabricate biliary stents must comply with biocompatibility and manufacturing regulations (ISO 10993 [30], ISO 13485 [31] and ISO 14971 [32]). In addition, the FDA has released guidance to specifically address the use of 3D printing for medical devices [33,34]. To date, experimental and pre-clinical studies on 3D-printed biliary stents are in short supply. In 2019, Boyer utilized a MakerBot fused deposition modeling (FDM) printer for their 15 F biliary stent [35]. Kim, in 2022, and Lee, in 2024, are the only others to have published studies on 3D-printed biliary stents; their polycaprolactone (PCL) stents, 12 F and 9 F, respectively, were also made with a modified FDM system [26,36].

While stereolithography (SLA) 3D printers have superior resolution to FDM 3D printers [37,38,39], the cytotoxicity of common photo-initiators such as trimethylbenzoyl-diphenylphosphine oxide (TPO) [40,41] in SLA resins has been a marked deterrent to the use of SLA 3D-printing for medical devices. As it were, ultraviolet (UV)-cured polymer composites have been widely used in dental applications for nearly 50 years [42]. In turn, beginning with Formlabs’ Dental SG resin in 2016, a number of medical grade SLA resins are now commercially available [43,44,45,46,47]. Many, if printed and post-processed accordingly, meet some or all of the requirements for use in a Class I medical device; a few can meet Class II requirements [48]. The progress we have seen with SLA resins in the last 5 years is a strong indicator that with the appropriate resin, printing parameters, and post-processing, there is potential for an SLA 3D-printed polymer to satisfy the mechanical demands and biocompatibility requirements for use in a biliary stent. Herein, we detail our efforts to design and fabricate an SLA 3D-printed polymeric biliary stent with a commercially available resin that addresses some of the shortcomings of current biliary stents while keeping Class II medical device regulations in mind.

## 2. Materials and Methods

### 2.1. Three-Dimensional Printing, Post-Processing, and Sterilization

A computer-aided design (CAD) model of an American Society for Testing and Materials (ASTM) D-1708 [49] micro-tensile sample was generated with Fusion 360 (version 2.0.18961, Autodesk, San Francisco, CA, USA). The micro-tensile sample’s .STL file was imported into PreForm software (version 3.15.2, Formlabs, Somerville, MA, USA) and prepared for printing with Durable (FD) or BioMed Durable (FBD) (Formlabs, Somerville, MA, USA) resin at 100 μm layer height on a Form 3 or Form 3B SLA printer (Formlabs, Somerville, MA, USA); samples were supported on the print platform with manually placed 0.3–0.4 mm touchpoints and mini-rafts. All samples were washed while attached to the print platform in isopropanol (>99.5%) in a Form Wash (Formlabs, Somerville, MA, USA), followed by air drying for 30–60 min at room temperature. Samples were then detached from the print platform, and the supports were removed. Next, samples were post-cured in a Form Cure (Formlabs, Somerville, MA, USA). The Form Cure was pre-heated, and samples were placed inside once the cure temperature was reached; samples were promptly removed from the Form Cure at the completion of the cure time. After samples were post-cured, they were sterilized via autoclave gravity cycle (121 °C) in sterilization pouches; samples were promptly removed from the autoclave at the completion of the cycle. Specific wash durations, post-cure settings, and autoclave durations for all post-processing and sterilization treatments tested can be found in Table 1 below.

### 2.2. Mechanical Characterization

FD and FBD micro-tensile samples were tested to failure on a Mark10 ESM 303 (Copiague, NY, USA) (*n* = number of samples, *n* ≥ 6 per post-processing treatment). Strain was calculated assuming a 20 mm length for the neck of the micro-tensile sample. Elastic moduli were determined from the slope of linear trendlines fitted to the stress–strain curve below the yield stress of each sample. Peak stress was calculated with the force applied (in N) when the micro-tensile sample failed, divided by the cross-sectional area of the neck of the micro-tensile sample (in m^2^). Statistical significance was determined via ANOVA single-factor analysis.

### 2.3. Cytotoxicity Testing

CAD models of cytotoxicity samples were generated in Fusion360. The cytotoxicity material sample had a conical media interface of 14.52 cm^2^ in compliance with ISO 10993-12 [50] for minimum material-sample–media interface (6 cm^2^/mL, Figure S1A). The FD cytotoxicity material samples (*n* = 3 per treatment) were printed as described in Section 2.1 and post-processed as Treatment 5 or Treatment 6 (Table 1).

FD cytotoxicity material samples were incubated with 5 mL/well DMEM without phenol red (Sigma Aldrich, MO, USA) in 6-well plates at 37 °C and 5% CO_2_ for 72 h (Figure S1B,C). In parallel, mouse fibroblasts (P5–P12; 3T3-L1, ATCC, Manassas, VA, USA) were cultured in DMEM without phenol red (Sigma Aldrich, MO, USA) at 37 °C and 5% CO_2_. After 72 h, cells were passaged and seeded in a 96-well plate (*n* = 5 per material sample, 100 μL media/well) and 12-well plates (*n* = 3 per material sample, 1 mL media/well) with a 3:1 mixture of media exposed to FD cytotoxicity samples and fresh media (containing the cells). All seeded plates were incubated at 37 °C and 5% CO_2_ for 48 h. At 28 h and 48 h, phase contrast photos of cells seeded in 12-well plates were taken on a Nikon TMS inverted microscope at 10× (*n* = 3 photos per well). 

A CellTox Green Cytotoxicity Assay (Promega, Madison, WI, USA) was performed after 48 h culture in material-exposed media for live/dead cell quantification in the 96-well plate. Maximum cytotoxicity controls were generated by adding a lysis buffer to designated wells. Following addition of the CellTox reagents, the plate was incubated in the dark at room temperature for 15 min. The plate was then read on a BioTek Synergy HTX (Agilent, Santa Clara, CA, USA) (ex 488, em 540). Statistical significance was determined via ANOVA single-factor analysis.

In addition, after 48 h in sample-exposed media, genomic DNA was isolated from the fibroblasts grown in 12-well plates using an SV Wizard kit (Promega, USA). The genomic DNA samples were stored at −20 °C and quantified within a week using a NanoDrop (ThermoFisher Scientific, Waltham, MA, USA). Statistical significance was determined via ANOVA single-factor analysis.

### 2.4. Biliary Stent Designs

**Figure 2:**
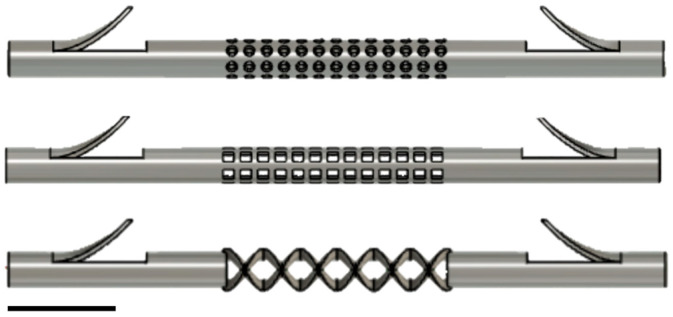
CAD models of biliary stent designs. **Top**: Circle stent, **Middle**: Square stent, **Bottom**: Weave stent. Scale bar = 10 mm.

Three biliary stent CAD models were generated with Autodesk Fusion360. All stents were 60 mm long with an outer diameter of 3.3 mm (10F), an inner diameter of 2.3 mm, and a perforated, 20 mm middle section (Figure 2). The stents were named “Circle stent” and “Square stent” per the shape used to create the perforations, and the third stent, “Weave stent” [51], given the appearance of the perforated section.

### 2.5. Computational Fluid Dynamics (CFD) of Bile Flow with Biliary Stents 

A CFD model of bile flow through the stents was generated using ANSYS Fluent 22.1 (Ansys, Canonsburg, PA, USA). To simulate a stent deployed at a juncture of the extrahepatic bile ducts, a stent was placed with its perforated section spanning a 30° junction of two flow paths (4 mm and 3.4 mm, respectively). Bile velocity and the pressure change in the duct from the inlet to outlet were simulated with a laminar model [52] and the following parameters: bile flow rate of 0.5 mL/min [52,53], bile viscosity of 1 mPa·s or 5 mPa·s [54,55], and bile density of 1000 kg/m^3^. This bile flow rate was selected based on a person weighing 200 lbs [56] and a bile production rate of 5 µL/min/kg [53]. Inflation layers were applied to the walls of the flow path. The number of mesh elements ranged from 400 K to 800 K per model. The initial condition for flow path velocity at the inlets and flow path outlet pressure was set to 0 m/s and 0 Pa, respectively.

### 2.6. Optimization of FD Stent Printing Orientation

The stent .STL files were imported into Formlabs PreForm software. Stents were then arranged at 0°, 30°, 45°, and 60° to the print platform and supported with manually placed 0.4 mm touchpoints. Stents were printed with Formlabs Durable resin at 100 μm layer height on a Formlabs Form 3 or 3B printer (*n* ≥ 3 per angle). Printed stents were removed from the print platform with their supports intact. They were then subjected to Treatment 1 or Treatment 2 (Table 1). After stents cooled to room temperature, the supports were removed, and stent dimensions were measured with electronic precision digital calipers. Statistical significance was determined via ANOVA single-factor analysis.

### 2.7. In Silico Stent 3-Point Bend Testing

Three-point bend tests of the stents were simulated using Ansys Static Structural (version 20.1, Canonsburg, PA, USA). The Ansys isotropic linear elastic material models were generated using a Poisson’s ratio of 0.43 (polypropylene) and the Young’s modulus, tensile yield strength, and tensile ultimate strength from the FD-Treatment 6 tensile tests. The stent CAD geometries with the flanges removed (to simplify the simulation) were imported as .step files; for the static structural set up, the ends of each stent were fixed, and a remote force (1 N to 10 N, 1 N increments) was applied to the outer face of the midpoint of each stent (Figure S2A). The total displacement and the equivalent (von-Mises) stresses were examined for each stent geometry at each applied force.

The meshes for the Circle and Square stents were generated using the same method. The physics preference, element order, and element size were set to nonlinear mechanical, linear, and 0.5 mm, respectively. Then, the outer face of the stent was selected, and mesh refinement was set to 3. The mesh method for the Weave stent was slightly different, due to the Weave stent’s more complex geometry. The physics preference, element order, and element size were the same as used for the Circle and Square stents; however, it was necessary to define a face sizing of 0.05 mm for the central 20 mm of the Weave stent (Figure S2B). 

### 2.8. Experimental Stent 3-Point Bend Testing

To observe the mechanical behavior of the stent designs and evaluate the accuracy of our FEA results, three-point bend tests were performed on an ESM 303 MTS (Mark-10, USA) with MEASURgauge Plus software. (version 2.1.0, Mark-10, USA We followed ASTM D790 and ASTM F2606 [57,58] but modified the testing protocol to accommodate for stent geometry. Due to the cylindrical geometry of the stents, the ends were fixed via clamps. Prior to testing stents, the bend test anvil was positioned just above the center of the first stent, and travel was set to zero; the anvil was returned to zero travel following each test. Stents were tested to failure (travel: 10 mm/min, force gauge: 5 samples/sec, Figure S2C).

## 3. Results

### 3.1. Effects of Post-Processing Modifications and Sterilization on Formlabs Durable Polymer Mechanical Properties

**Figure 3:**
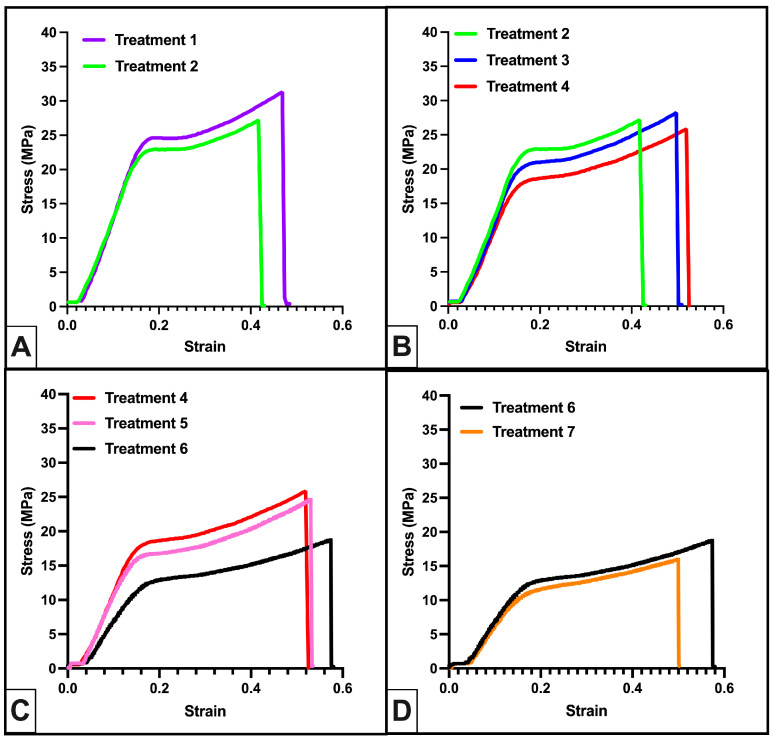
Improved elasticity of Formlabs durable polymer through modifications to Form Wash and Form Cure durations and temperatures. (**A**) Stress–strain curves for FD micro-tensile samples post-processed as recommended (Treatment 1) and with sterilization by gravity autoclave (Treatment 2); *n* ≥ 8. (**B**) Stress–strain curves for FD micro-tensile samples post-processed with recommended Form Wash time and Form Cure time but reduced Form Cure temperatures; *n* ≥ 7. (**C**) Stress–strain curves for FD micro-tensile samples post-processed with adjustments to both Form Wash duration and Form Cure time and temperature; *n* ≥ 5. (**D**) Stress–strain curves for FD micro-tensile samples with modified Form Wash time, modified Form Cure time and temperature, and sterilized by 30 or 60 min gravity autoclave; *n* ≥ 5. Stress–strain curves shown are from a representative sample from each set of micro-tensile samples.

We initially evaluated the mechanical properties of Formlabs Durable with Formlabs recommended post-processing: 20 min IPA wash in Formlabs Form Wash and 60 min at 60 °C in a Formlabs Form Cure along with the impact of subsequent gravity cycle autoclaving (Treatment 1 and Treatment 2). Autoclave sterilization had little effect on the elastic moduli (E) or peak stress of the materials (Table 2, Figure 3A). Our FD-Treatment 2 biliary stent models fractured upon modest handling and/or bending, so we evaluated changes to post-processing parameters that could improve FD elasticity. 

We first investigated the effects of reducing the Form Cure temperature (Treatment 3 and Treatment 4). We found that elasticity increased modestly with decreasing Form Cure temperature (Table 2, Figure 3B). Next, we evaluated a reduced Form Cure time at a reduced temperature or a combination of increased Form Wash time with the reduced Form Cure time and temperature (Treatment 5 and 6). Both treatments improved elasticity over Treatment 4, but Treatment 6 yielded a significant reduction in the elastic modulus (161.57 ± 12.16 MPa vs. 108.38 ± 8.81 MPa, *p* < 0.01). Finally, we confirmed that a shorter gravity autoclave cycle (Treatment 7) did not alter the elasticity (E = 104.89 ± 12.6 MPa vs. E = 108.38 ± 8.81 MPa, Figure 3D).

Upon Formlabs’ release of FBD, advertised as potentially compatible with short-term (<24 h) contact with tissue and dentin applications [59], we compared its mechanical properties after undergoing Treatment 6 to FD-Treatment 6. We found FBD-Treatment 6 was significantly less elastic (152.67 ± 16.71 MPa vs. 104.89 ± 12.6 MPa, *p* < 0.0001) and slightly stronger (22.58 ± 1.69 MPa vs. 18.95 ± 2.01 MPa, *p* < 0.005) than FD-Treatment 6. Per our expectation that the stent material needed to be highly elastic, we did not evaluate FBD-Treatment 6 any further.

### 3.2. Cytotoxicity Testing of FD with Modified Post-Processing Parameters and Autoclave Sterilization

**Figure 4:**
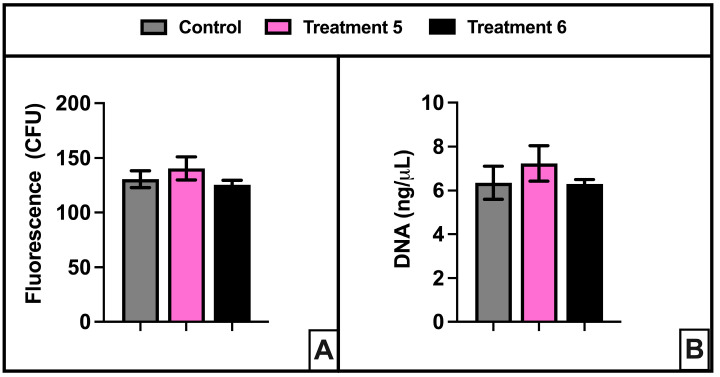
No cytotoxicity from media exposed to FD-Treatment 5 or FD-Treatment 6 in mouse fibroblasts. (**A**) Cell death was minimal and not significantly different from the DMEM-only-exposed controls in sample-exposed media. (**B**) The concentration of fibroblast DNA per well was not negatively affected by culture in sample-exposed media.

Qualitative evaluation of stents printed in FD-Treatment 5 or FD-Treatment 6 supported further testing and evaluation of those materials. In keeping with our goal of generating stents that conform to Class II device regulations, we ran preliminary ISO 10993-based cytotoxicity studies on FD-Treatment 5 and FD-Treatment 6 samples. Our results showed no significant increase in cell death at 48 h due to culture with FD-Treatment 5- or FD-Treatment 6-exposed media (Figure 4A). In addition, images of the fibroblasts (Figure S4) and DNA quantification at 48 h (Figure 4B) indicated no ill-effects on cell morphology from culture in sample-exposed media.

### 3.3. CFD Model of Bile Flow at an EHBD Juncture with and without Stents

**Figure 5:**
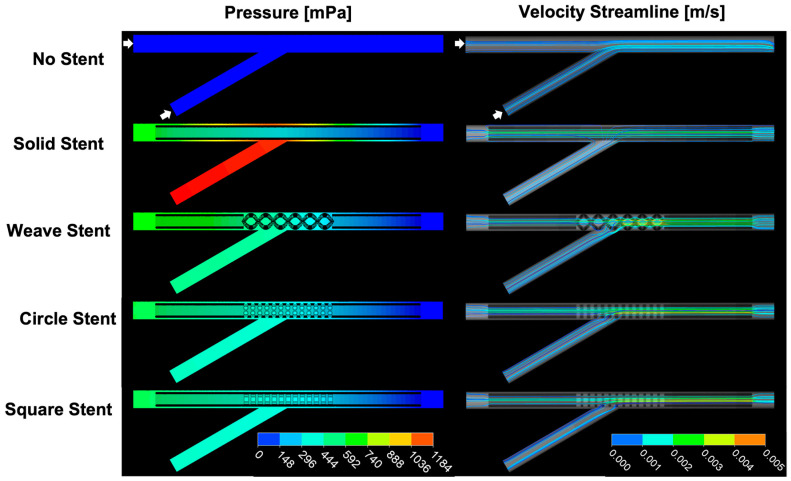
Predicted pressure gradients and velocity streamlines in EHBDs when biliary stents are deployed spanning an EHBD juncture. Results shown for bile viscosity of 1 mPa·s. Arrows indicate EHBD inlet for simulation.

The pressure difference (ΔP) from proximal to distal in a stented bile duct can be used to evaluate the effectiveness of a biliary stent [52,60,61]. In healthy patients, pressures in the common bile duct ranges from 400 Pa to 1000 Pa [60,62]; in patients suffering from cholestasis, pressure in the common bile duct can spike above 3000 Pa [63,64]. Meanwhile, bile viscosity is relatively stable [52,54,65]. Simulations of bile flow at a bile duct juncture with bile flow rates and viscosities typically seen in fasting, healthy patients [54,65,66] demonstrated that all the stents with perforated middle segments reduced the pressure build up at the junction seen with a solid stent (Figure 5, Table S4). The Weave, Circle, and Square stents all reduced the predicted pressure build up by roughly 50% (Table S4). 

### 3.4. Effects of Print Orientation on Print Platform

We evaluated the effects of the print angle (relative to the print platform) as accuracy and precision are necessary in the production of medical devices, particularly those devices that can be tailored to an individual patient. We found the Form 3/3B to be both accurate and precise at 45° and 60° but less so at 30° and 0°. Stents printed at 45° or 60° showed minimal deviations from the CAD stent dimensions (Figures S4–S6 and Table S2). Alternatively, stents printed at 0° were consistently longer than designed and required individual stent flushing with IPA prior to the Form Cure because of residual resin in the lumens (Figure S4, orange arrow). Both 0° and 30° stents had frequent instances where, on one end of the stent, one side of the stent or both sides were distorted (Figures S4–S6, End Views).

### 3.5. Accuracy of In Silico Stent 3-Point Bend Tests

**Figure 6:**
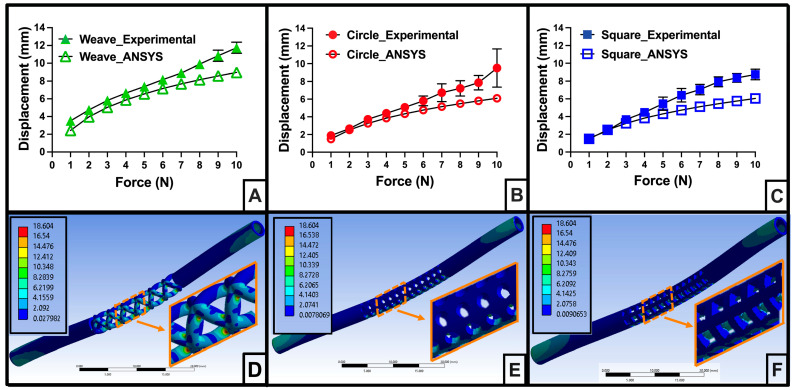
Accuracy of in silico stent 3-point bend test and predicted peak stresses. (**A**–**C**) Stent displacements during experimental and simulated 3-point bend tests (*n* ≥ 6 for experimental results). Weave stent 3-point bend test displacement results were accurate, i.e., within 15% of experimental 3-point bend test results, for applied forces from 3 N to 7 N. The Circle stent in silico results were accurate from 2 N to 5 N, and the Square stent design were only on target for 3 N and 4 N. Only the Weave stent reached the target displacement (7.76 mm) in the accurate range of the simulation. (**D**–**F**) Predicted peak stresses at 5 N for each of the stent designs. The peak stress locations, when the simulation is accurate, indicate where the stent is likely to stretch or fracture. Scale bar in (**D**–**F**) is 20 mm. Predicted peak stress in (**D**–**F**) ranged from near zero to (blue) to 18.6 MPa (red) in increments of ~2 MPa.

Three-point bend test simulations in Ansys using mechanical properties from FD-Treatment 6 (E = 104.89 ± 12.6 MPa, yield stress = 12.06 ± 0.38 MPa, and peak stress = 18.9 MPa ± 2.01 MPa) predicted stent displacements up to 8.98 mm, 6.10 mm, and 6.05 mm for Weave, Circle, and Square stent models, respectively, as force applied was increased from 0 N to 10 N (Figure 6A–C). Next, we performed 3-point bend tests on FD-Treatment 6 stents to evaluate the accuracy of our in silico 3-point bend test (Table S2). The stents tended to be displaced further at a given force during the experimental bend tests than predicted by the simulations, and this became more pronounced above 5N force applied (Figure 6A–C). 

### 3.6. In Silico Predicted Peak Stresses in FD-Treatment 6 Stents

A bend angle of 30° is the most we expected a biliary stent would experience when deployed; that equates to a displacement of 7.76 mm for our 60 mm long stent (Figure S7). From our experimental 3-point bend test data, we identified the forces needed to displace each stent design 7.76 mm. For the Weave stent, 5 N–6 N was needed to achieve a bend of 7.76 mm. Alternatively, it took over 8 N for the Circle stent and over 7 N for the Square stent to bend that distance.

We then utilized finite element analysis (FEA) in Ansys to predict the peak stress that each stent design would experience if similar forces were applied (Table S3). Figure 6D–F shows the peak stress simulation results for each stent at 5 N.

Given the complex stent geometry, percent errors < 15% between experimental displacement and simulated displacement were our target [67,68]. The Weave stent percent errors for the forces applied from 3 N to 7 N were 13.8% and under. At 5 N, with an error of 11.2%, the predicted peak stress of 18.6 MPa for the Weave stent is likely a reliable estimate. Alternatively, the percent errors for the Circle and Square stent simulations were only <15% up to 5 N. With 8 N (error = 24.0%) and 7 N (error = 27.5%) needed to bend the Circle and Square stents, respectively, the predicted 21.8 MPa and 13.7 MPa peak stresses are not accurate estimates of the material behavior at those forces.

## 4. Discussion

The goals of this work were to (1) determine if it is feasible to utilize a commercially available SLA resin to fabricate potentially FDA-compliant biliary stents, (2) generate 3D-printable biliary stent designs that permit bile flow at a duct junction and are compatible with placement via catheter during ERCP, and (3) develop an accurate simulation of polymeric stent behavior to aid in evaluating novel stent designs and/or novel materials. In this discussion, we will review our results and consider them in the context of other relevant studies.

### 4.1. Commercial SLA Resin Feasibility

We selected FD resin because results from our previous studies indicated it was both flexible but tough [69]. Formlabs describes Durable resin as “simulating the strength and stiffness of polyethylene” [70]. Polyethylene is commonly used in microcentrifuge tubes for the processing and storage of biological samples; it is autoclavable and biocompatible. If printed FD is indicated to have similar mechanical properties to polyethylene, we presume it would have a similar chemical composition once polymerized and post-processed. Knowing the exact chemical composition is not necessary; our interests lie in its ultimate mechanical properties and biocompatibility. Thus, if the photoinitiators in FD resin are fully consumed and/or removed during printing and post-processing, and the resin is fully polymerized, it might be a suitable material for an FDA Class II device.

We also wanted a material that could be steam sterilized via gravity cycle autoclave. Sterilization is critical for medical devices. Steam sterilization is the primary method of sterilization utilized worldwide [71,72]; alternatives like ethylene oxide (EtO) and irradiation can pose health risks and are not always accessible [73].

In turn, we anticipated that an extended IPA wash would remove residual photoinitiators, and the additional exposure to heat during autoclaving would increase the polymerization of the polymer reducing monomers free to migrate from the polymer [74,75,76,77]. While other studies have utilized high-performance liquid chromatography or Fourier transform infrared (FTIR) spectroscopy to demonstrate the completion of polymerization [74,78], as this was a preliminary study, we did not quantify polymerization. We took the lack of cytotoxicity as a promising result and plan to include FTIR spectroscopy in our material characterization in the future.

We must note that, in June 2023, Formlabs released the BioMed Durable (FBD) resin. It is a “new formulation”; when one looks at the SDS for FBD composition, it shows four ingredients, all “Trade Secrets” [79]. The four ingredients are in similar proportions to the four ingredients in FD [80]: Acrylate Monomer(s), Methacrylate Monomer(s), photoinitiator(s), and Urethane Dimethacrylate, but the photoinitiator(s) in FBD is listed as <1%. Our preliminary evaluation of FBD’s mechanical properties, when post-processed and autoclaved similarly to FD, showed it was significantly stiffer. This, coupled with the cost of FBD relative to FD ($429/L vs. $199/L), warranted tabling further evaluation of FBD for inclusion in these studies. We recognize that the elasticity of FBD could be improved with post-processing protocol modifications and may address those options in the future.

Since we began work on these studies, several other companies have released resins that could meet the material needs for a polymeric biliary stent. Until recently, only Form 2 permitted ‘open source’ printing, and now, the upgrade to enable ‘open source’ on Form 3 is costly. We have initiated studies utilizing a printer with broader resin compatibility in hopes of being able to cost-effectively evaluate available resins that have mechanical properties in our range of interest.

### 4.2. Design and Fabricate an SLA 3D-Printed Stent That Permits Bile Flow at a Duct Junction and Could Be Placed during ERCP

When a stent is needed to traverse the junction of the left and right hepatic ducts, as is often the case in biliary hilar malignancies, the American Society of Gastrointestinal Endoscopy recommends bilateral stenting with plastic stents or SEMs [81,82]. Our stent designs would allow, if the patient’s pathophysiology supported it, placement of just one stent in lieu of two at the junction of bile ducts, which could reduce cost to the patient and decrease the difficulty of the procedure. CFD simulations of our stents placed at a duct junction versus a solid stent deployed at a duct junction showed that perforations in the stent at the duct junction would reduce the pressure buildup by roughly 50%. Of note, the CFD simulations were performed with an initial duct pressure of 0 Pa, yielding pressure changes of <1.5 Pa for bile viscosity of 1 mPa·s and <6.0 Pa for bile viscosity of 5 mPa·s. Pressures in healthy common bile ducts range from 400 Pa to 1000 Pa [60,62]; bile viscosity is stable [52,54,65], and fasting bile flow rate is small [53]. Were we to have used, e.g., 500 Pa as the initial pressure in our calculations, changes in pressure due to stent placement and/or stent architectures, while not insignificant, would have been difficult to visualize (Section 3.2, Figure 5). Of the three stent designs we tested in this study, we believe the Weave stent to be the best candidate for continued evaluation. The larger holes in the perforated section of the stent should better facilitate drainage from the adjoining duct and have less tendency to clog.

The stents also needed to be dimensionally accurate and tolerate sterilization as quality control is highlighted in the FDA guidelines for 3D-printed medical devices [34], and sterilization is necessary in medical device manufacturing [83]. FD-Treatment 6 stents printed on a Form2 SLA printer at a 60° angle to the print platform had minimal variations from the CAD stent dimensions (Figures S4–S6, Table S2). We combined a shorter Form Cure time with autoclaving to obtain a sterile but more elastic product. This did reduce the material peak stress, but we do not intend the stents to experience loads approaching peak stress.

In recent years, several groups have 3D-printed fully perforated biliary stents. Kim et al. [26] and Lee et al. [36] used modified FDM printers to fabricate polycaprolactone (PCL) biliary stents. While neither group disclosed their sterilization method prior to implantation, due to PCL’s material properties, it can be deduced that EtO sterilization was utilized. Boyer et al. [35] 3D-printed a mold for a polyvinyl alcohol-based biliary stent and sterilized their stent via immersion in 70% ethanol.

**Figure 7:**
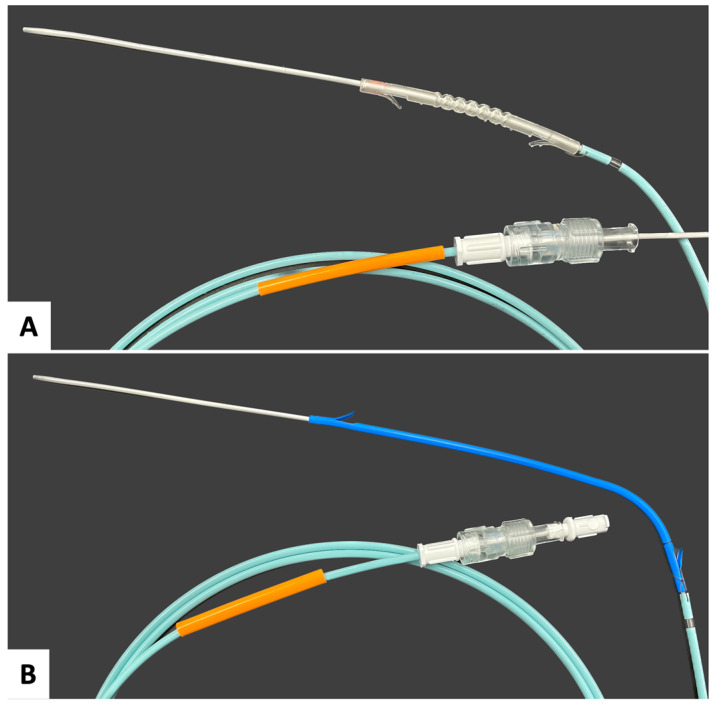
Stents loaded onto a standard biliary stent deployment catheter. (**A**) Weave stent (60 mm, 10 Fr) on catheter; (**B**) Flexima (Boston Scientific) (100 mm, 8.5 Fr) on catheter.

Of the three, only Lee’s was compatible with catheter deployment. Figure 7A shows our Weave stent (10 Fr) loaded on a catheter; Figure 7B shows a commercially available catheter-deployed plastic stent (8.5 Fr) for comparison. Overall, the stents fabricated with Durable that underwent Treatment 6 were consistent in size, readily tolerated gravity cycle sterilization, and qualitatively appeared suitable for the target application.

### 4.3. Accurate In Silico Stent 3-Point Bend Test

We aimed to generate an in silico test to evaluate new polymeric biliary stent designs. To ensure a polymeric biliary stent does not permanently deform or fracture when deployed, the peak stress that the stent experiences from bending forces should not exceed the yield stress of the polymer [84,85,86]. A plastic biliary stent is typically bent no more than 30° when deployed; this equates to a displacement of 7.74 mm for a 60 mm long stent. For a complex geometry and simulating with a non-standard material, we were looking for <15% relative error between our experimental displacement and our simulated displacement. At the forces needed to bend our stents between 7 mm and 8 mm, only the Weave stent’s simulated displacement was within 10% relative error of the experimental displacement (Table S2). The greater degree of accuracy could be due to the more refined meshing needed for the central portion of the Weave stent. In turn, the in silico 3-point bend test predicted that the peak stresses within the center portion of the Weave stent at applied forces of 5 N–6 N (needed for 7–8 mm displacement) would exceed the yield stress of FD-Treatment 6 (Section 3.1, Table 2) by over 5 MPa (Table S3).

## 5. Conclusions

Our results indicate the Formlabs Durable resin, when post-processed with a longer IPA wash and autoclaved, could be considered for use in an FDA Class II medical device. We found that biliary stents compatible with catheter deployment could be fabricated with FD resin on Formlabs SLA printers. These stents may address some of the drawbacks of currently available biliary stents in that they are relatively inexpensive to fabricate, they withstand autoclave sterilization, and they can be readily tailored to address patient-specific anatomical features. They could also, per the mid-stent perforations, enable placement of only one stent in lieu of two when stenting is required at a duct junction. However, our stents would likely have limited patency similar to the plastic stents on the market as they are comparable in dimension. Lastly, and perhaps most importantly, our in silico 3-point stent bend test with FD-Treatment 6 revealed that while our stents appeared adequately flexible when we handled them, they would potentially experience peak stresses that could cause structural damage when deployed. Moving forward, we will evaluate FD copolymers and modifications to post-processing to improve the elasticity of FD-Treatment 6.

## 6. Patents

1. Thomas, J. L.; Patel, S. G. Patmas Weave Stent. US D987,826 S, 30 May 2023.
2. Thomas, J. L.; Patel, S. G. Patmas Weave Stent. EU 008 883 797-0001.

## Figures and Tables

**Table 1:** Formlabs Durable post-processing and sterilization combinations. Treatment 1 is the Formlabs Durable post-processing recommended by Formlabs.

Treatment	IPA Wash (min)	Air Dry (min)	UV Post-Cure Time (min)	UV Post-Cure Temperature (°C)	Autoclave Cycle	Autoclave Time (min)
1	20	30–60	60	60	-	-
2	20	30–60	60	60	gravity	60
3	20	30–60	60	50	gravity	60
4	20	30–60	60	40	gravity	60
5	20	30–60	10	40	gravity	60
6	60	30–60	10	40	gravity	60
7	60	30–60	10	40	gravity	30

**Table 2:** Mechanical properties of Formlabs Durable polymer after modifications to post-processing and sterilization. See Table 1 for treatment protocols. *n* ≥ 6 per treatment.

Treatment	Elastic Modulus (MPa)	Yield Stress (MPa)	Peak Stress (MPa)
1	191.38 ± 8.72	21.43 ± 1.28	34.37 ± 3.76
2	177 ± 10.67	19.51 ± 1.32	27.64 ± 2.19
3	185.7 ± 10.77	17.97 ± 1.08	26.93 ± 2.83
4	161.57 ± 12.16	16.63 ± 1.42	25.77 ± 2.08
5	148.99 ± 11.09	14.00 ± 0.66	22.81 ± 1.11
6	104.89 ± 12.6	12.06 ± 0.38	18.95 ± 2.01
7	108.38 ± 8.81	9.96 ± 0.79	18.37 ± 1.71

## Data Availability

The original contributions presented in the study are included in the article/Supplementary Material, further inquiries can be directed to the corresponding author.

## Supplementary Materials

The following supporting information can be downloaded at: https://www.mdpi.com/article/10.3390/polym16141978/s1. Figure S1: Cytotoxicity Samples and Media Exposure. Figure S2: In Silico and Experimental 3 Point Bend Test Method. Figure S3: Morphology of 3T3-Fibroblasts during cytotoxicity test with FD-Treatment 5 (center) and FD-Treatment 6 (right)-exposed media. Figure S4: Effect of Print Angle on Weave Stent Dimensional Accuracy. Figure S5: Effect of Print Angle on Circle Stent Dimensional Accuracy. Figure S6: Effect of Print Angle on Square Stent Dimensional Accuracy. Figure S7: Maximum Stent Displacement when 60 mm Stent is Bent 30°. Table S1: Summary of the Effect of Print Orientation on Dimensional Accuracy. Table S2: Summary of In Silico and Experimental 3 Point Bend Testing Results. Table S3: Summary of In Silico 3 Point Bend Test Peak Stress Predictions. Table S4: CFD Predicted Pressure Difference Across Bile Duct Junction with and without Stents.
