# Supplementary figures and images for: Towards a Customizable, SLA 3D-Printed Biliary Stent: Optimizing a Commercially Available Resin and Predicting Stent Behavior with Accurate In Silico Testing
Source: Polymers (Basel). 2024 Jul 11;16(14):1978. doi: 10.3390/polym16141978 (PMC11280906; doi:10.3390/polym16141978)

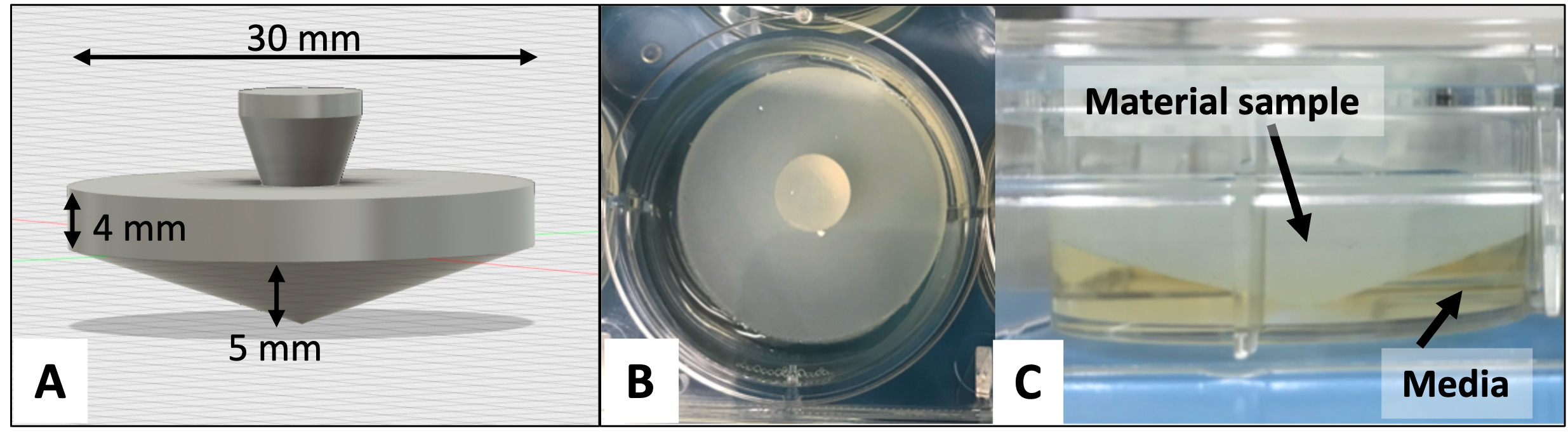

Supplement: Supplementary file 1 [file polymers-16-01978-s001.zip › Figure S1_Cytotoxicity Media Exposure Method.tiff]

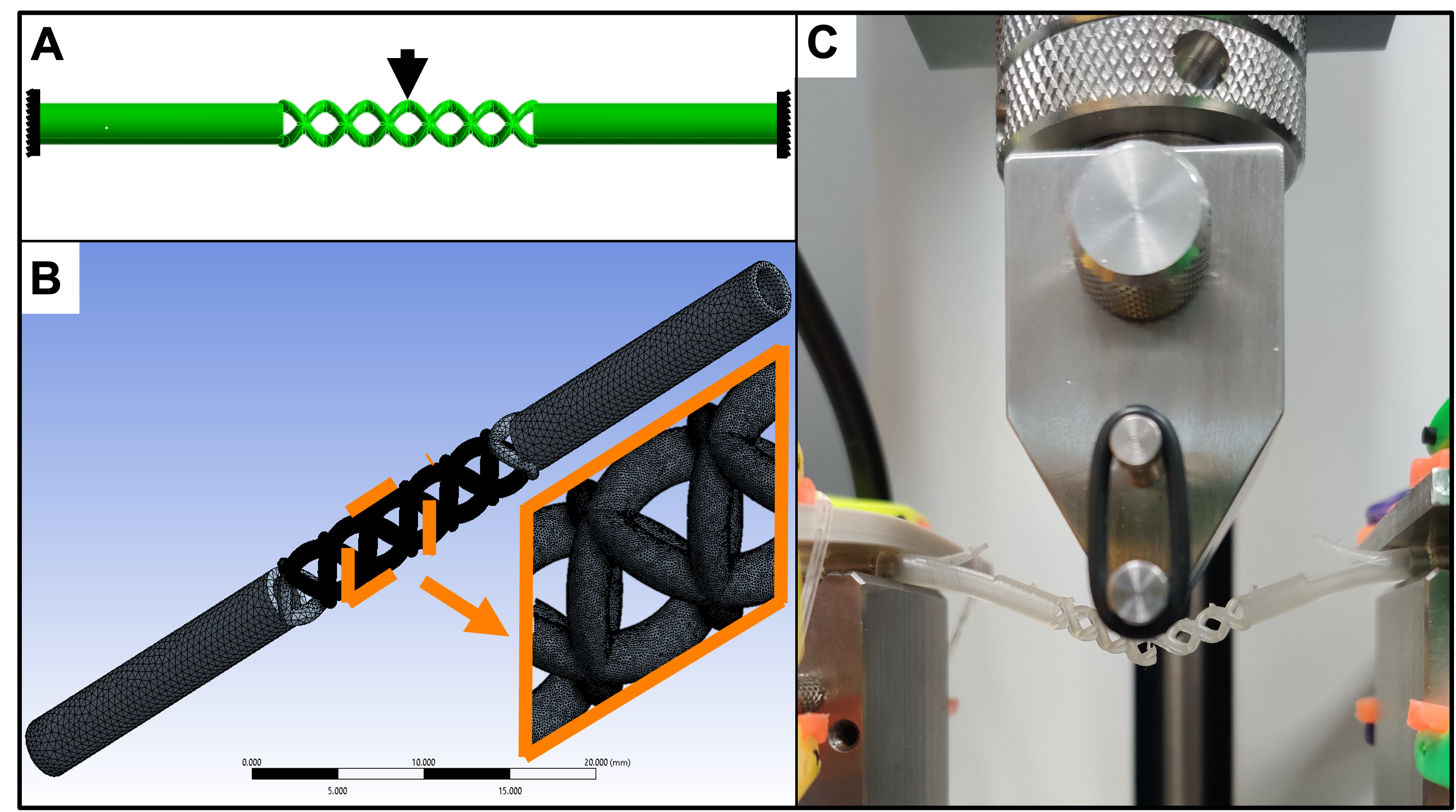

Supplement: Supplementary file 1 [file polymers-16-01978-s001.zip › Figure S2_Bend Test_Methods.png]

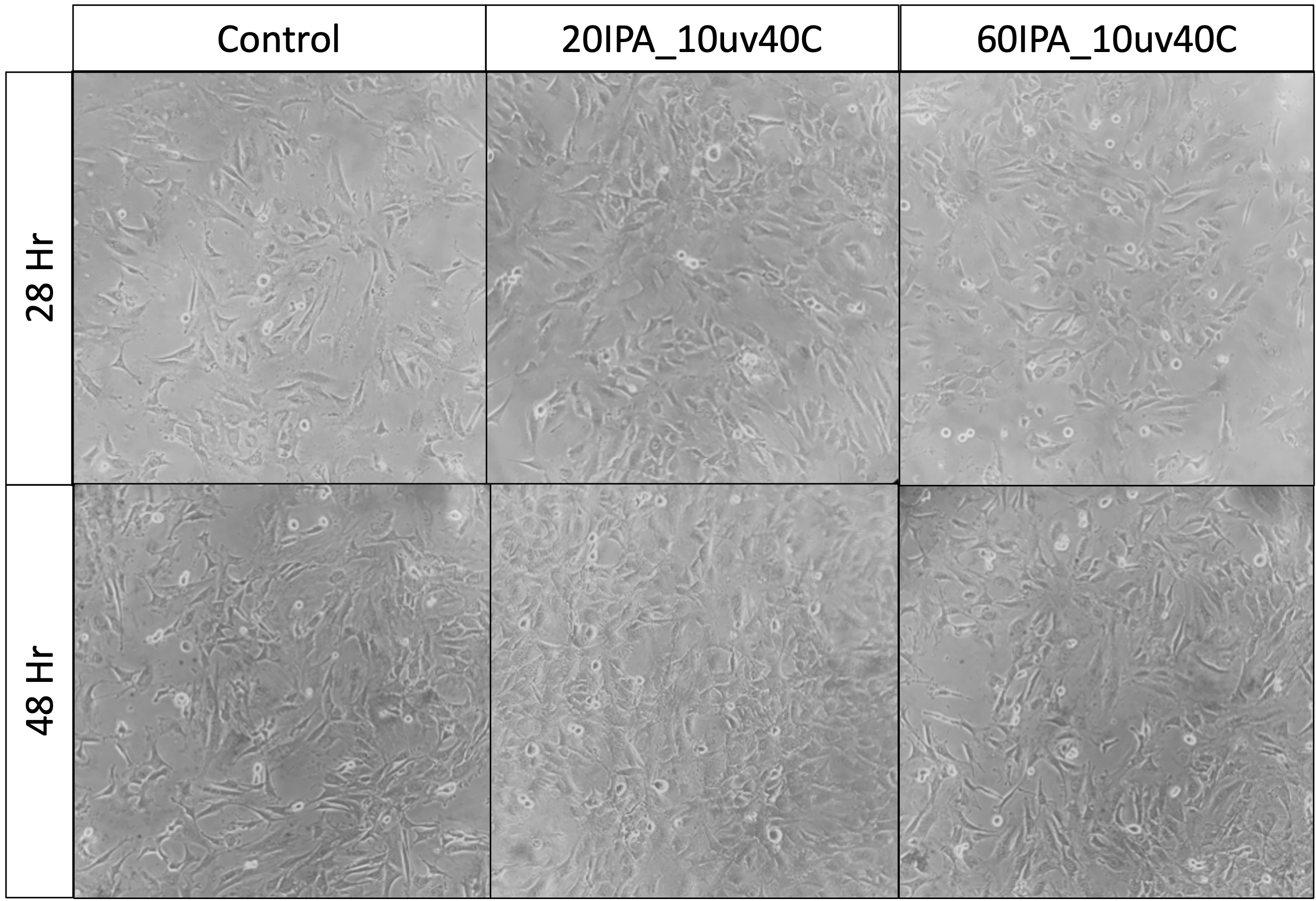

Supplement: Supplementary file 1 [file polymers-16-01978-s001.zip › Figure S3_Dur_3T3 cytotoxicity_cell pics.png]

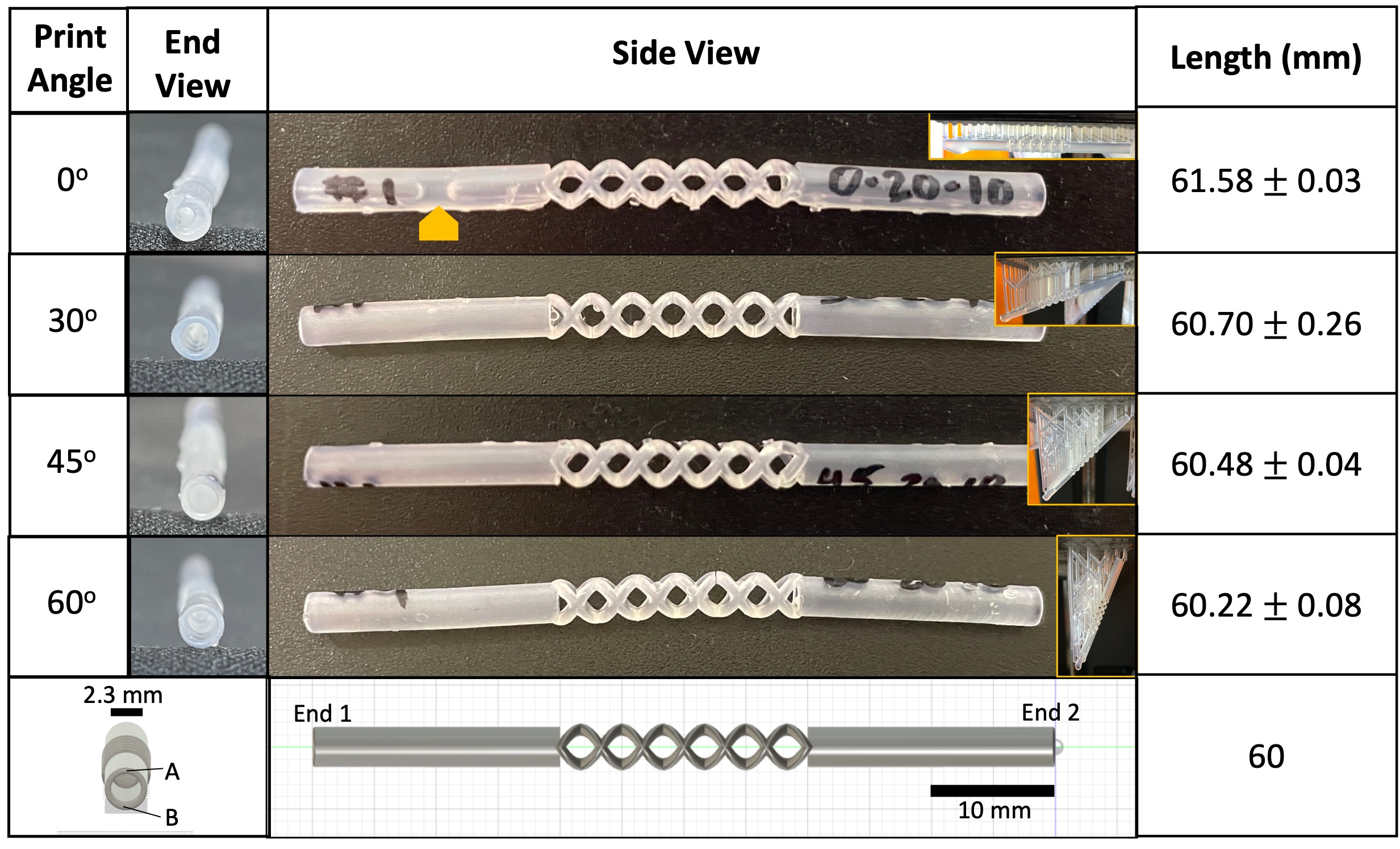

Supplement: Supplementary file 1 [file polymers-16-01978-s001.zip › Figure S4_Weave Stent Print Angle.tiff]

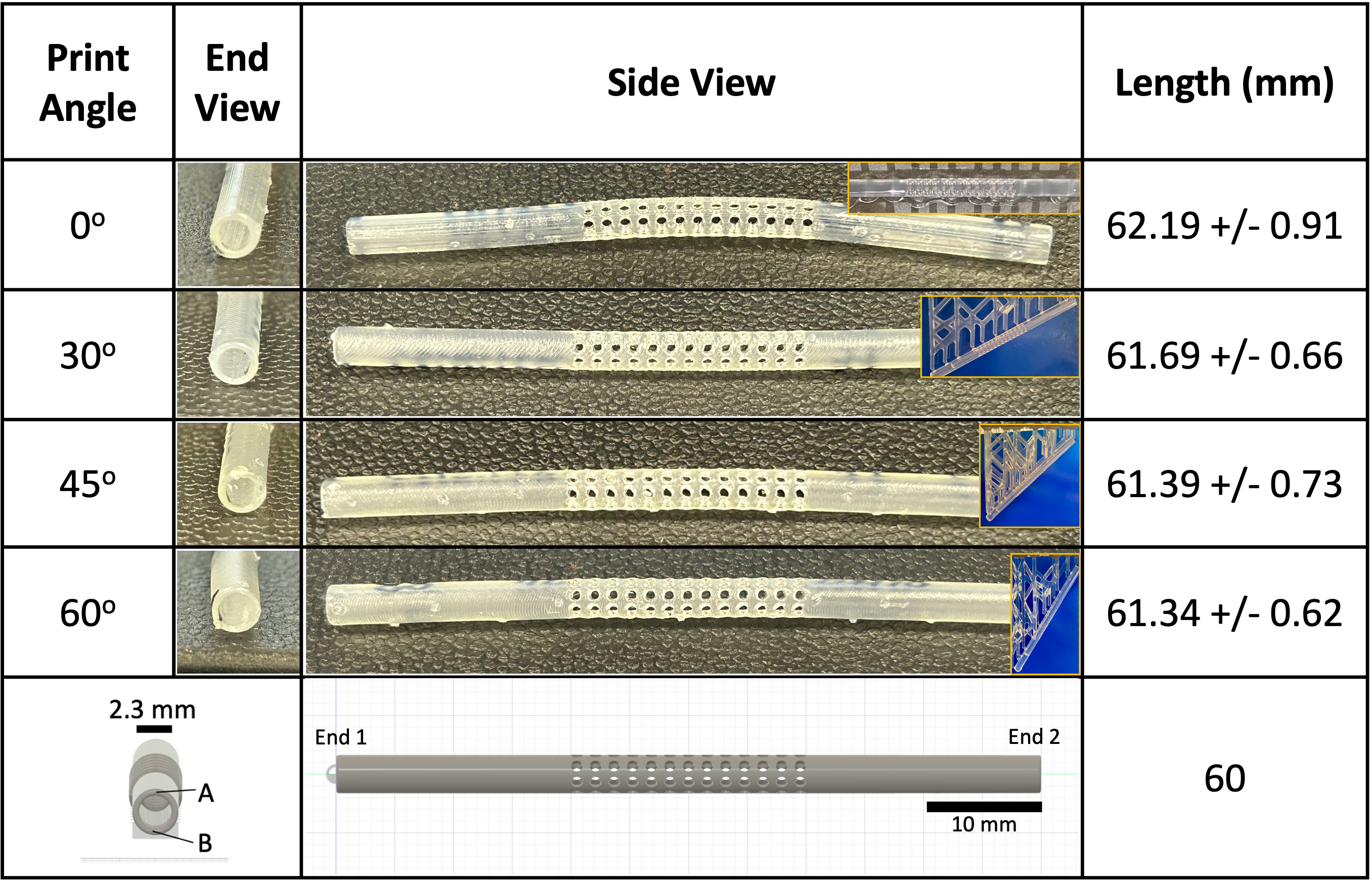

Supplement: Supplementary file 1 [file polymers-16-01978-s001.zip › Figure S5_Circle Stent Print Angle.png]

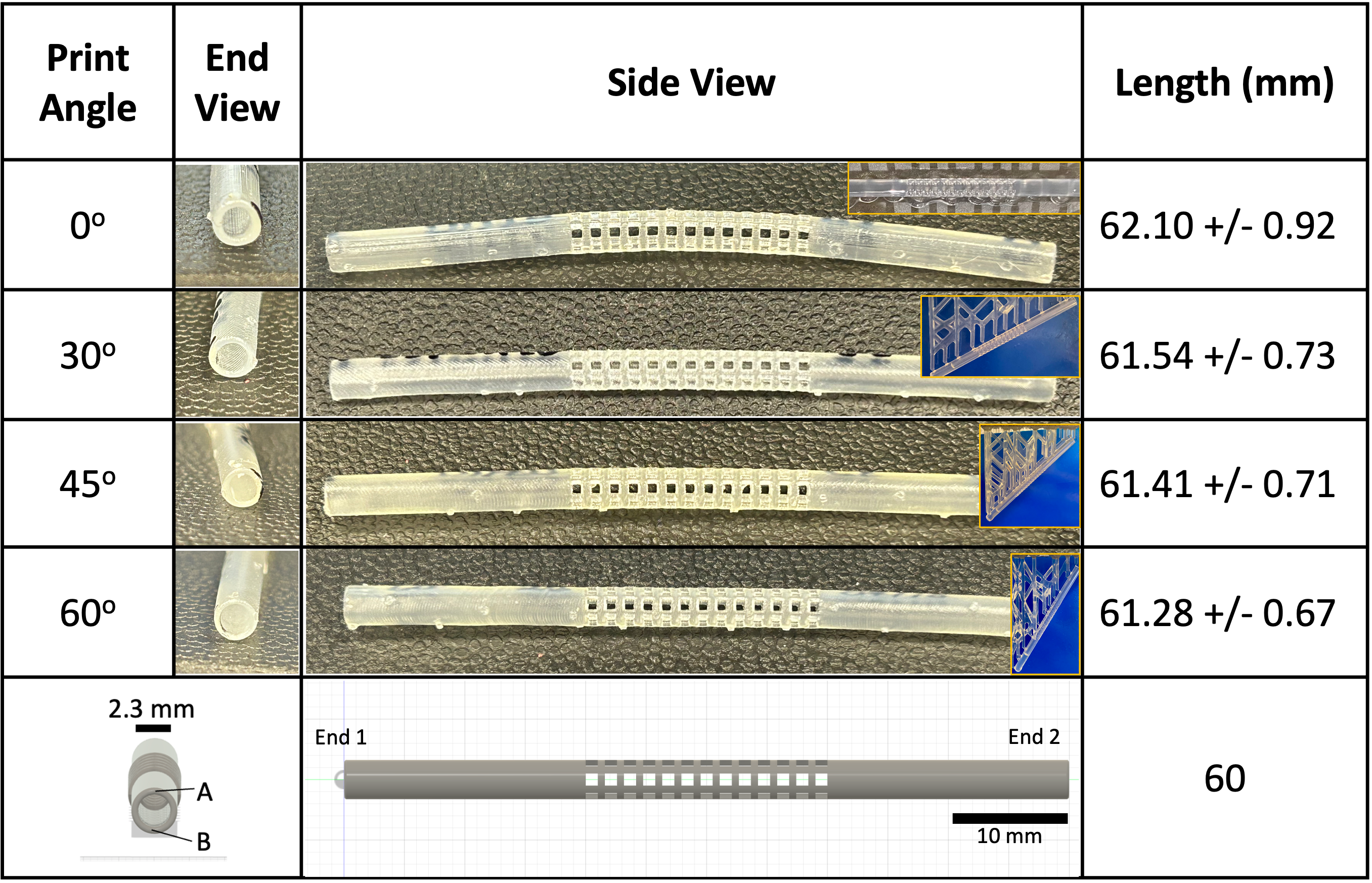

Supplement: Supplementary file 1 [file polymers-16-01978-s001.zip › Figure S6_Square Stent Print Angle.png]

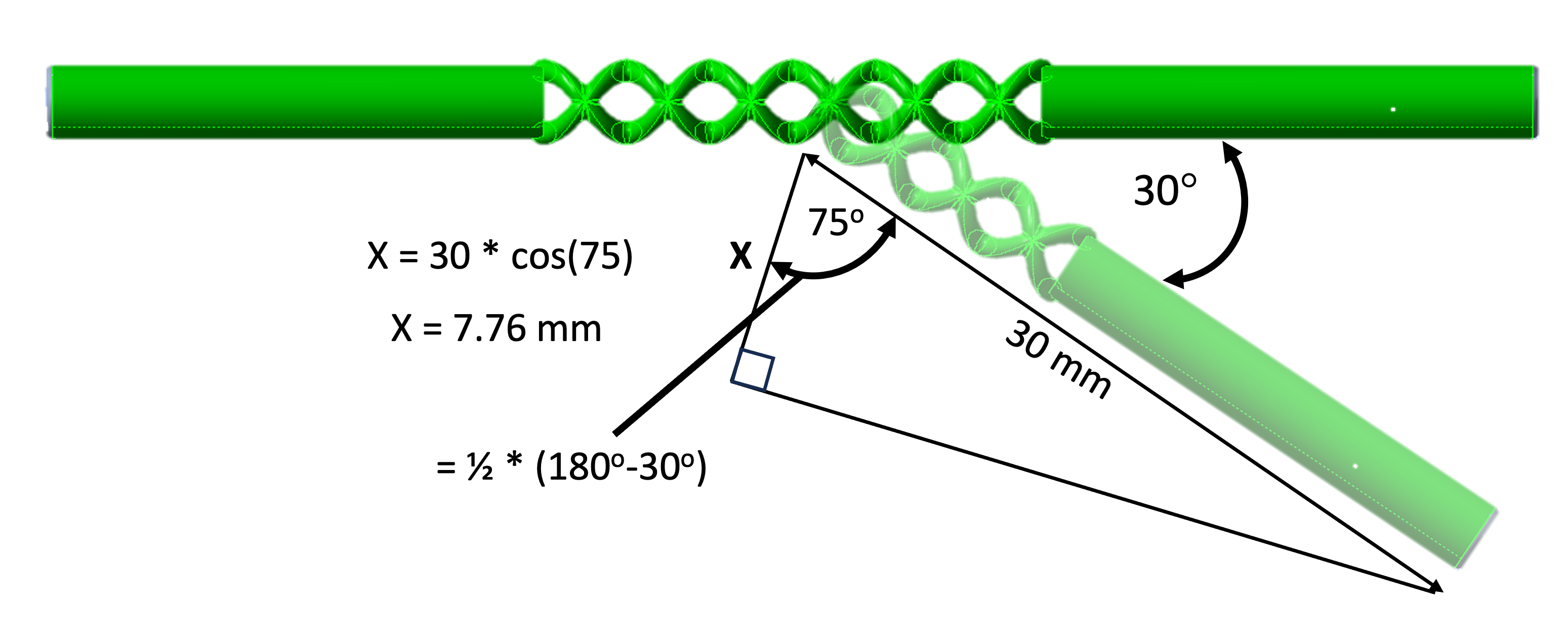

Supplement: Supplementary file 1 [file polymers-16-01978-s001.zip › Figure S7_Max displacement w30degBend.png]

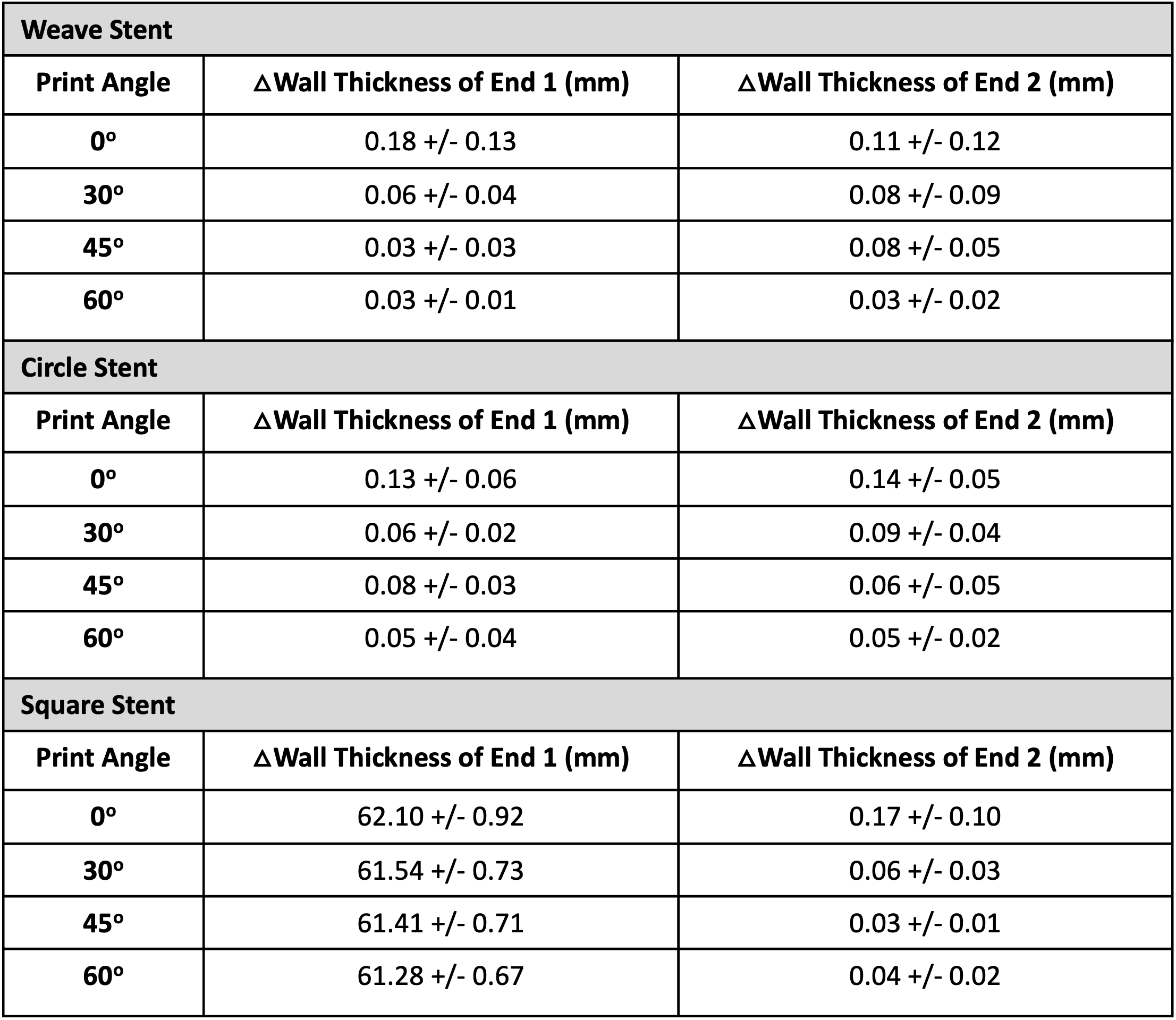

Supplement: Supplementary file 1 [file polymers-16-01978-s001.zip › Table S1_Print Orientation Effect.png]

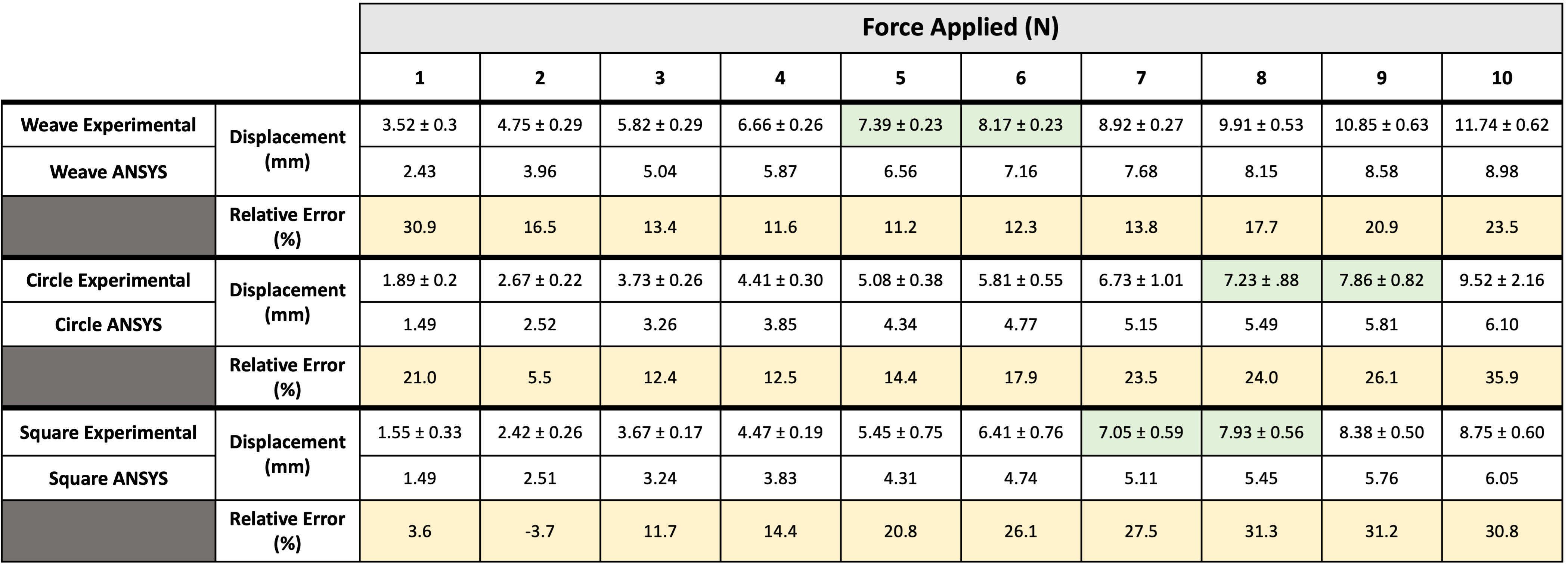

Supplement: Supplementary file 1 [file polymers-16-01978-s001.zip › Table S2_Bend Test_Exp vs ANSYS.png]

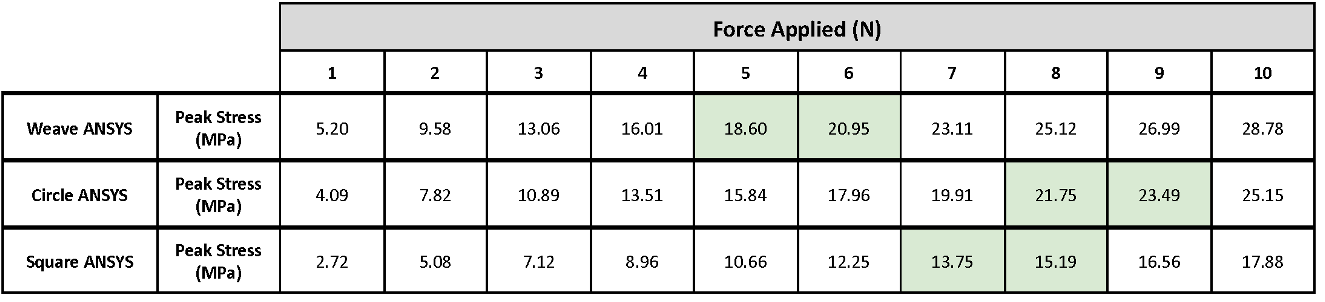

Supplement: Supplementary file 1 [file polymers-16-01978-s001.zip › Table S3_Predicted Peak Stress_3 stent designs.png]

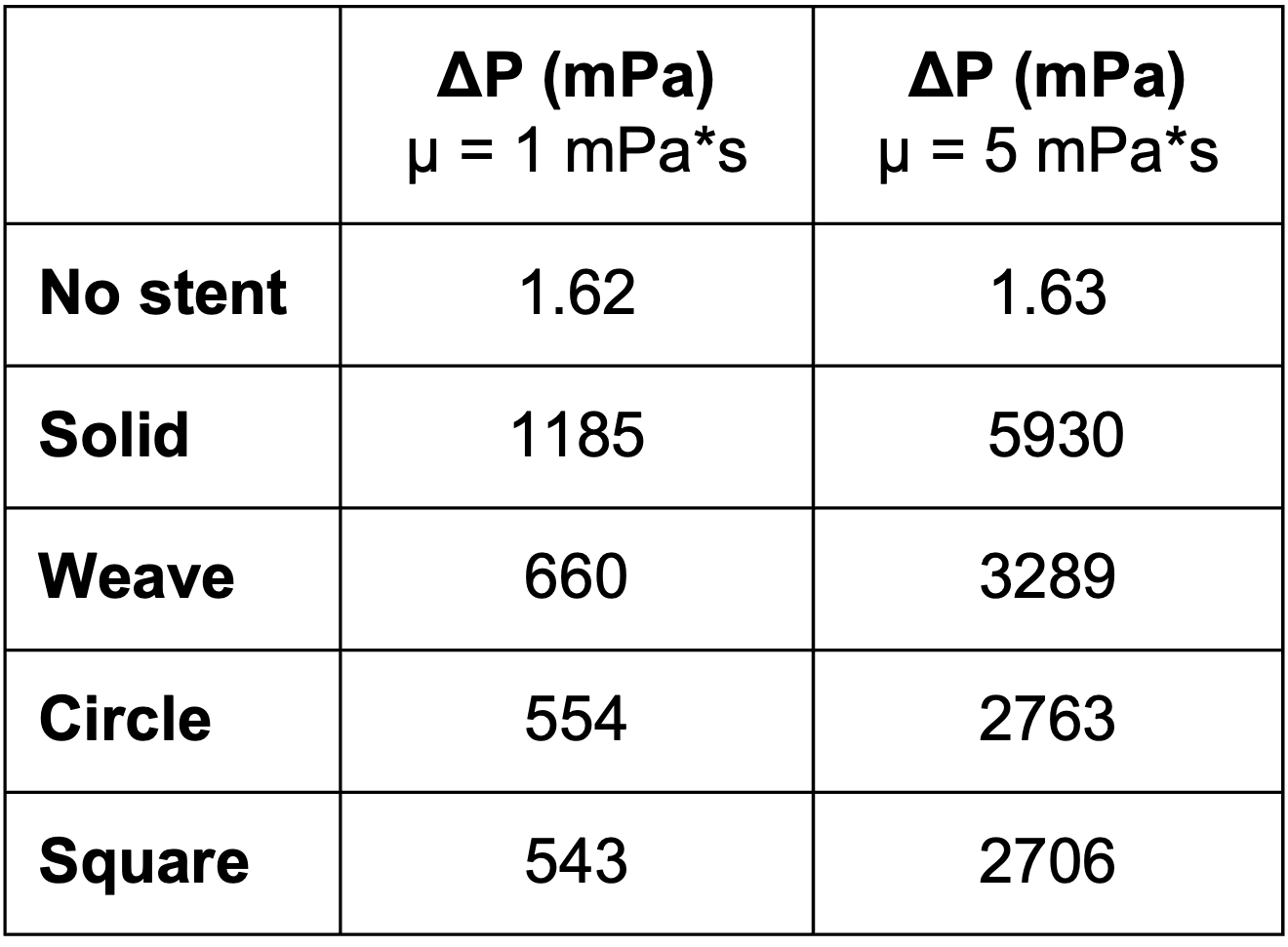

Supplement: Supplementary file 1 [file polymers-16-01978-s001.zip › Table S4_CFD results.png]
